# Mimetic host shifts in an endangered social parasite of ants

**DOI:** 10.1098/rspb.2012.2336

**Published:** 2013-01-22

**Authors:** Jeremy A. Thomas, Graham W. Elmes, Marcin Sielezniew, Anna Stankiewicz-Fiedurek, David J. Simcox, Josef Settele, Karsten Schönrogge

**Affiliations:** 1 Department of Zoology, University of Oxford, Oxford OX1 3PS, UK; 2 Centre for Ecology and Hydrology, Maclean Building, Wallingford OX10 8BB, UK; 3 Department of Invertebrate Zoology, Institute of Biology, University of Bialystok, Swierkowa 20B, 15-950 Białystok, Poland; 4 Laboratory of Social and Myrmecophilous Insects, Museum and Institute of Zoology, Polish Academy of Sciences, Wilcza 64, Warszawa, Poland; 5 Department of Community Ecology, UFZ Helmholtz Centre for Environmental Research, 06120 Halle (Saale), Germany

**Keywords:** chemical mimicry, host specificity, butterfly, conservation, *Maculinea*, *Phengaris*

## Abstract

An emerging problem in conservation is whether listed morpho-species with broad distributions, yet specialized lifestyles, consist of more than one cryptic species or functionally distinct forms that have different ecological requirements. We describe extreme regional divergence within an iconic endangered butterfly, whose socially parasitic young stages use non-visual, non-tactile cues to infiltrate and supplant the brood in ant societies. Although indistinguishable morphologically or when using current mitochondrial and nuclear sequence-, or microsatellite data, *Maculinea rebeli* from Spain and southeast Poland exploit different *Myrmica* ant species and experience 100 per cent mortality with each other's hosts. This reflects major differences in the hydrocarbons synthesized from each region by the larvae, which so closely mimic the recognition profiles of their respective hosts that nurse ants afford each parasite a social status above that of their own kin larvae. The two host ants occupy separate niches within grassland; thus, conservation management must differ in each region. Similar cryptic differentiation may be common, yet equally hard to detect, among the approximately 10 000 unstudied morpho-species of social parasite that are estimated to exist, many of which are Red Data Book listed.

## 1. Introduction

To set meaningful priorities in conservation and for practical remedies to succeed, it is vital to ascertain whether the threatened morpho-species named in Red Data lists are likely to consist of more than one cryptic species [1,2] or functionally distinct genotypes. In theory, regional (co-)adaptations may be amplified in closely coupled biological systems [3], such as obligate mutualisms [4] and host–parasite arms races [5]. In the case of insect–insect interactions, both parties may be susceptible to strong selection within small spatial scales, even between neighbouring landscapes [6,7]. Thus, unexpected subsets of host specificity, rapid evolution and cryptic speciation are an emerging feature of insect-parasitoid studies [8], and apparently exist, possibly in extreme forms, among the estimated 100 000 morpho-species of poorly studied insects that interact with ants (myrmecophiles) [9].

DNA analysis transformed biologists' ability to detect cryptic species within the described morpho-species [10,11], and with a burgeoning array of reference sequences available (e.g. Consortium for the Barcode of Life), an increasing number of species complexes are being identified [2]. Nevertheless, commonly used, affordable techniques may be insensitive to detecting differentiation that has arisen recently or from selection on one or a few genes that affect the phenotype in major ways [12–14]. *Maculinea* (large blue) butterflies exemplify this problem: all six recognized morpho-species are iconic flagship insects, long identified as global conservation priorities [15–17], that possess attributes associated with cryptic speciation [2], including socially parasitic young stages that use non-visual, non-tactile cues, including chemical and acoustical mimicry, to infiltrate and exploit ant societies [15–19]. Recent analyses of mitochondrial- (COI, COII) and nuclear sequence data (EF1α, wingless), as well as microsatellite data, suggest that each morpho-species of *Maculinea* that has a predatory lifestyle contains cryptic lineages, but recognized cuckoo species (species that are fed directly on regurgitations by ants; see the electronic supplementary material, figure S1*e*) could not be distinguished [15,20–22], despite their closer integration and local coevolution with ant societies and, typically, more extreme host specificity [9,23,24].

We studied the regional divergence that nevertheless appeared to exist within the endangered cuckoo butterfly *Maculinea rebeli* (Hirschke), which was itself indistinguishable from a close relative, *Maculinea alcon* (Denis & Schiffermüller), in recent molecular studies [20,21]. Prior to 1991, each of these congeners was classed as globally vulnerable [25], but uncertainty about their taxonomic status led to exclusion from subsequent lists. It is not disputed that *Ma. rebeli* and *Ma. alcon* are distinct ecotypes (or putative ecospecies) which inhabit different ecosystems, xerophytic and moist grassland, respectively, and exploit different plant and *Myrmica* species [9,22]. Both are extreme specialists whose respective larvae feed initially on the flowerheads of *Gentiana cruciata* and *Gentiana pneumonanthe* on typical sites, a distinction widely used, including here, to classify the two types. Larvae then infiltrate *Myrmica* ant colonies in their final instar, where they live for 11–23 months and acquire more than 98 per cent of their ultimate biomass (see the electronic supplementary material, figure S1) [9]. They achieve this transition by abandoning their host plant and secreting simple cocktails of hydrocarbons that resemble the chemical signatures of *Myrmica* grubs sufficiently well to trick foraging workers of any *Myrmica* species to ‘rescue’ the mimic and carry it into the underground brood chambers [9,18]. However, although each caterpillar is adopted indiscriminately by the first forager to encounter it [9], each *Myrmica* species whose nest it enters represents not only a different food but also a different enemy, chemical template to mimic [26] and living environment for 92–96% of the intruder's life: unsurprisingly, caterpillars typically survive with the single, or occasionally sibling, model ant species that they mimic best [9]. Thus, within colonies of the model host species, the intruding larvae successfully compete with the ant brood for worker attention and are soon fed (and rescued) preferentially by the nurse ants (see the electronic supplementary material, figure S1*e*), a subterfuge that is achieved by synthesizing additional hydrocarbons shortly after adoption that more precisely mimic their host *Myrmica* species (but other *Myrmica* species less) [27]. By contrast, caterpillars carried into nests of other *Myrmica* species suppress their secretions and rely on the passive acquisition of their host's gestalt odour for social integration [27]. Acquired camouflage alone, however, is insufficient to survive periods of stress or deprivation, when nurse ants become discriminatory and xenophobic [28].

We noticed that populations of *Ma. rebeli* in southwest Europe and Poland appear to exploit very different species of *Myrmica*, *Myrmica schencki* and *Myrmica sabuleti*, respectively [9,29]: ants whose chemical recognition profiles differ more than any other known pairs of *Myrmica* species [26] and which occupy different niches within grassland. We therefore studied the exclusivity of host specificity that has evolved in each region by measuring survival both in natural populations and in the laboratory. We then devised behavioural experiments to assess the social status achieved by Spanish and Polish larvae after infiltrating the two host ant societies, and also identified the mechanism responsible for host specificity by analysing the mimetic chemicals secreted by pre- and post-adoption larvae from each region. Finally, we described the key attribute of the niche occupied by each ecotype (or cryptic species) of this endangered butterfly, which provides the essential knowledge for their future conservation [16].

## 2. Material and methods

### (a) Measuring host specificity in natural populations

Host specificity was measured by comparing the proportions of caterpillars that were adopted into different *Myrmica* nests with the proportions that survived to adulthood or pupation. Data were obtained from three populations for 5 consecutive years near Panticosa in the Spanish Pyrenees [9,30] and in one population for 4 years near Przemyśl, southeast Poland [22]. The proportion of larvae adopted by different ants was estimated by baiting beneath stratified random samples of gentians and by counting the number of eggs on each plant [30,31]. Previous work had shown that there was no difference in egg or larval survival on gentians growing in different ant territories, nor in the ratio of larvae retrieved from beneath plants by different ant species: the first *Myrmica* worker to encounter a larva retrieved it, and where two species overlapped, there was no bias in retrieval towards one species [30,32]. The distribution of the egg population on gentians is, therefore, an accurate surrogate for the distribution of the final-instar population entering nests of each *Myrmica* species [30,32]. Adult estimates of *Ma. rebeli* were obtained by recording eclosing individuals along stratified transects across sites, and identifying the nest after confirming that it contained an empty pupal case [30,31]. Additional data were obtained by excavating all *Myrmica* nests near gentians along stratified transects that had supported known densities of eggs the previous year and by counting the pupae they contained. No mortality has been recorded in pupae in *Myrmica* cells before eclosion [30–32].

**Figure 1.**
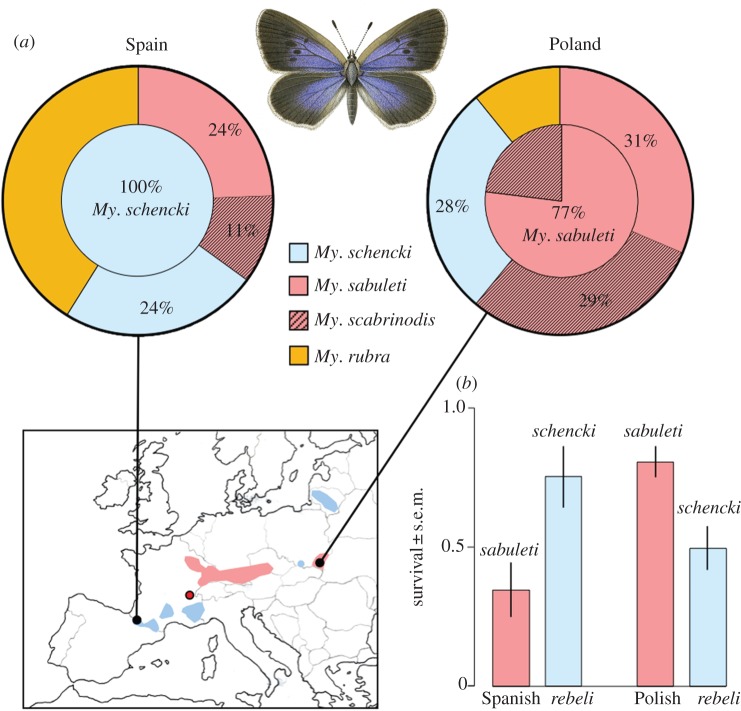
Host specificity of *Maculinea rebeli* in Spain and southeast Poland. (*a*) Field survival: outer circle, egg distribution (=larval adoption, see §2*a*) in different *Myrmica* ant territories (*n* = 2859 Spain, 102 Poland); inner circle, ant species where *Ma. rebeli* survived to pupae or adults (*n* = 148 Spain, 548 Poland). Map: blue, *My. schencki* recorded as sole host; pink, *My. sabuleti* primary host; red circle, source of laboratory test ants in Alps. (*b*) Larval survival after 17 days in paired laboratory ant colonies set from the same naive French source nests.

A map of regional host specificity (figure 1) was compiled from our published results [9,29–32] supplemented by additional field data. The distributions are considered to be near complete for Poland [22,29], the French and Spanish Pyrenees and southern Alps [30–32], but the northern Alps and Massif Central were less comprehensively sampled and may contain greater complexity in host use. Host use in Italy, Hungary and central Switzerland was not mapped.

### (b) Laboratory experiments of host specificity

Host specificity from the two regions was measured using naiïve laboratory *My. schencki* and *My. sabuleti* colonies, collected from the Jura, east France, midway between the Pyrenees and Przemyśl in a landscape lacking the butterfly (figure 1). Six nests of each species were excavated and divided into subcolonies, maintained on a standard diet in Brian nests [33]. After 6 to 8 weeks acclimatization, more than 100 *G. cruciata* flower spikes were randomly collected from the Polish and Spanish sites, and the resultant final-instar larvae were used within 12 h of leaving their foodplant to establish experiments (§2*b–d*). First, larval survival was measured in 22 laboratory cultures, each containing 50 workers and five ant larvae, established from the stock nests (six pairs of *My. schencki*, five pairs of *My. sabuleti*). A total of 123 *Ma. rebeli* larvae from Poland and 97 from Spain were introduced in groups of seven so that each set of cultures contained matching pairs of colonies, each derived from the same stock nest. Larvae that died in the first 7 days were replaced, and total survival in each nest was recorded after 17 days. Statistical analysis was conducted by paired two-tailed *t*-test.

### (c) Social status achieved in natural and unnatural host colonies

**Figure 2.**
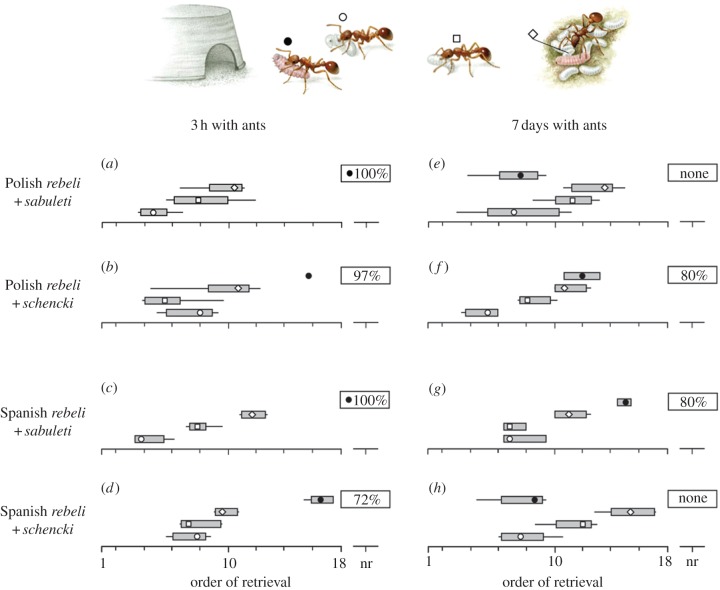
Status achieved by *Maculinea rebeli* within natural and unnatural *Myrmica* host societies. (*a–h*) The order that disturbed workers rescued ant brood or butterfly larvae 3 h and 7 days after adoption. Each replicate involved a choice between kin ant pupae (open circle), large kin ant larvae (open square), small kin ant larvae (open diamond), *Ma. rebeli* larvae (filled circle). Boxplots show means of median orders of rescue (symbol), 25–75% quartiles (box), and first and last individuals (tails); ‘nr’ = per cent *Ma. rebeli* larvae not retrieved by ants after 30 min. All treatments showed significant differences in the order in which items were retrieved (Kruskal–Wallis, *H* = 10.38–25.84, d.f. = 3, *p* = 0.016 to < 0.001), with fewer *Ma. rebeli* rescued than ant brood in (*a–d*,*f* and *g*) (*z* = −12.83 to −46.66, *p* < 0.001). After 7 days with their natural hosts (*e,h*), *Ma. rebeli* were rescued first equal with kin pupae (Mann–Whitney *W* = 38.5, *p* = 1.000; *W* = 24.0, *p* = 0.5309, respectively), significantly ahead of kin larvae (*p* = 0.024, 0.027). See the electronic supplementary material, table S1 for full statistical tests.

We assessed the social status achieved by each form of *Ma. rebeli* within colonies of each *Myrmica* species in a standard bioassay [23] that involved perturbing laboratory ant colonies and recording the order in which the ants' own brood or the mimetic caterpillars were rescued. Groups of five butterfly larvae from each region were adopted into matching colonies of naïive French *My. schencki* and *My. sabuleti*, using separate replicate nests to those used in §2*b*. Every test colony also contained five brood items each of kin ant pupae, large and small larvae, making a total of 20 immature individuals and 20 workers per replicate. Cultures were established in 413 cm^2^ boxes containing a small moist sponge pad beneath an inverted 6 cm diameter saucer with a notched entrance, under which the ants gathered their brood and *Ma. rebeli* (figure 2). Three hours after the *Ma. rebeli* caterpillars had been introduced, we perturbed the experimental colonies by uncovering the brood chamber and relocating it over another pad nearby; we then recorded the order in which the nurse ants rescued their 15 brood items or the five *Ma. rebeli* and carried them into the new nest (figure 2). The same experiment was repeated 7 days later, which represents a sufficient period for *Ma. rebeli* caterpillars to attain their maximum potential integration with a host society, yet remaining a similar size to when first adopted, i.e. the same size or smaller than the *Myrmica* pupae and large larvae [23,32]. The number of replicates for each ant–butterfly combination tested varied owing to a paucity of ant pupae and butterfly deaths (especially with unnatural hosts): *n* = 8 (figure 2*a,b*), *n* = 6 (figure 2*c,e*), *n* = 5 (figure 2*d,h*), *n* = 4 (figure 2*f*) and *n* = 3 (figure 2*g*). Fisher's exact tests were used to ascertain differences in the probability of a class of item being retrieved or abandoned by worker ants after perturbations. We also made three types of non-parametric analysis of the rank order in which chosen items were retrieved, within or between treatments: Kruskal–Wallis to establish whether ants rescued items randomly or selectively; Wilcoxon to test for changes in the order of selected items after *Ma. rebeli* had lived for 7 days with the ants compared with the initial 3 h; Mann–Whitney to test for differences in the order in which ant brood or butterfly caterpillars were selected within each of the eight combinations of ants and butterflies shown in figure 2. In addition to Mann–Whitney analysis, we used a randomization procedure whereby ranks were assigned at random for each trial twice. We recorded the difference in median between the two draws and repeated the procedure 10 000 times providing a frequency distribution for differences in medians to arise without selection. We then compared the observed differences in median between the item classes and assessed their likelihood to occur at random (see the electronic supplementary material, table S1).

### (d) Analysis of surface semio-chemicals on *Maculinea rebeli* larvae

To test whether observed regional differences in *Ma. rebeli*'s host specificity could be explained by variation in mimetic semio-chemicals, we tracked the changing chemical profiles of caterpillars from each region when reared with each ant, from uncontaminated pre-adoption final instars (see the electronic supplementary material, figure S1*b*), to individuals 6 weeks after adoption (see the electronic supplementary material, figure S1*e*), and finally to the latter after isolation from ants for 5 days, which allows acquired semio-chemicals to dissipate and prompts the hungry caterpillars to release their own secretions [27]. Hexane extracts of surface chemicals were obtained [18,26,27] from: (i) unparasitized *My. schencki* and *My. sabuleti* on our study sites in the Pyrenees, Poland and the naive ants in France that had not experienced *Ma. rebeli* (figure 1; *n* = 5 workers from five nests of each ant species per locality); (ii) eight batches per region of pre-adoption final-instar *Ma. rebeli* larvae sampled after leaving *G. cruciata* before contact with ants (see the electronic supplementary material, figure S1*b*; *n* = 5 larvae per batch, ∑40 individuals for each type of *Ma. rebeli*); (iii) *Ma. rebeli* larvae after living 6 weeks with naiïve French ants (see the electronic supplementary material, figure S1*e*), *n* = 5 (Spanish + *schencki* and Polish + *sabuleti*), *n* = 3 (Spanish + *sabuleti* and Polish + *schencki*); and (iv) *Ma. rebeli* larvae reared as in (iii), then isolated from ants and kept unfed and singly in sterile conditions for 5 days; *n* = 5 (Spanish + *schencki*), *n* = 4 (Polish + *schencki*), *n* = 3 (Spanish + *sabuleti* and Polish + *sabuleti*).

The chemical and statistical analyses of extracts followed an established protocol [26,27]. *Maculinea* extracts were concentrated to 20 μl, ant workers to 50 μl and 2 μl of every sample were analysed by gas chromatography with mass spectrometric detection using a HP 5890II gas chromatograph and HP 5971A mass selective detector, and ultra-high purity helium as the carrier gas with 10 psi column head pressure. Mass spectral data were acquired in full scan mode over 40–600 *m*/*z*. Mass chromatograms were initially screened for hydrocarbons by examining the selected ion chromatogram of *m*/*z* = 57. The chromatogram was integrated at a threshold value of 12 (HP integrator) to obtain the areas under the peaks measuring the total ion count. With each sequence of samples, we also analysed alkane standards (*n*-C20–*n*-C36), and the position of each peak within that range in a sample was calculated as an equivalent chain length (ECL) [26]. Mass chromatograms were inspected to ensure that they were free of gross interferences and that peaks of interest, such as branched and straight alkanes and alkenes, were chromatographically distinct and symmetrical. We excluded peaks that were column bleed, siloxanes or phthalate plasticizers as indicated by a characteristic abundant ion at *m*/*z* 149. Peaks of interest were tentatively identified by a combination of ECL number and inspections of their full scan mass spectra and matching with the NIST-97/08 mass spectral database.

For statistical analysis, the area under each peak was expressed as the proportion of the sum of all peaks in the chromatogram [26]. Samples were compared using multivariate and non-parametric multi-dimensional scaling on the ranks of the Bray–Curtis similarities [34]. The extent of a final lack of fit was assessed by a STRESS statistic [26] before pairwise differences between species and treatments were assessed using an analysis of similarities [35] in Primer-e v6. We used the average pairwise distance between groups, and assessed two averages with a two-sample *t*-test, to compare differences in the shift of similarities between groups.

### (e) *Myrmica *niches on* Maculinea rebeli* sites

Baits were placed under 223 flowering *G. cruciata* plants in Spain to record the species of *Mymica* foraging around them [31]. Vegetation structure (height) was measured using Stewart's direct method [36] at four diagonal points 5 cm from each plant. Species' niches were compared using two-tailed *t*-tests having confirmed normality of the data.

## 3. Results

### (a) Host specificity

In three *Ma. rebeli* populations over 5 years in the Spanish Pyrenees, we found that eggs were laid indiscriminately [37] on *G. cruciata* growing in the territories of four species of *Myrmica*, yet 100 per cent of adults emerged from *My. schencki* nests the following summers, despite only 24 per cent of the larval population being adopted by that ant (figure 1*a*; *z* =−96.81, *p* = <0.001). By contrast, for 4 years near Przemyśl, Poland, 28 per cent of *Ma. rebeli* larvae were adopted by *My. schencki*, but no adult emerged from their nests (*z* =−9.66, *p* = <0.001). Instead, 77 per cent of adults emerged from *My. sabuleti* nests and the remainder from *Myrmica scabrinodis*, a chemically similar [26] close sibling to *My. sabuleti* [38] (survival with *My. sabuleti* > *My. scabrinodis*; *z* =−3.85, *p* = <0.001). In Poland, we observed *My. schencki* workers eject mutilated *Ma. rebeli* larvae from the nests into which foragers had retrieved larvae a few hours earlier, with each corpse showing clear signs of attack by their putative hosts.

Well-fed captive ants are more tolerant of intruders than in the wild [28]. Nevertheless, we obtained a similar pattern of differential survival by *Ma. rebeli* within 17 days of introduction to standardized laboratory *My. sabuleti* or *My. schencki* cultures established from naive colonies from France (figure 1*b*; Spanish butterfly ≠ Polish butterfly survival with *My. schencki*, *t*_5_ = 2.51, *p* = 0.054; Spanish butterfly ≠ Polish butterfly survival with *My. sabuleti*, *t*_4_ = 4.10, *p* = 0.015).

### (b) Social status of caterpillars in natural and unnatural host species' colonies

*Maculinea rebeli* larvae did not integrate with their host society in the first hours after their adoption, being the last items to be chosen by workers on the rare occasions they were rescued after colony perturbation by exposure to light (see figure 2*a–d* and the electronic supplementary material, table S1). As expected [23], workers generally selected their pupae ahead of their large larvae and retrieved both in preference to small ant larvae. However, after a week with their natural hosts, *Ma. rebeli* from Spain and Poland were chosen equal first with the kin pupae of *My. schencki* and *My. sabuleti*, respectively, and significantly ahead of the smaller ant larvae (see figure 2*e,h* and the electronic supplementary material, table S1). But when each was reared with its unnatural ant host, just 20 per cent of week-old butterfly larvae were rescued, and these were afforded lowly status, ranking well below small ant larvae (see figure 2*f,g* and the electronic supplementary material, table S1). Even this may overestimate integration, because many *Ma. rebeli* larvae were killed in unnatural host *Myrmica* nests, and we perhaps tested the least maladapted individuals in the most socially accepting colonies.

### (c) Analysis of model and mimetic chemical profiles

**Figure 3.**
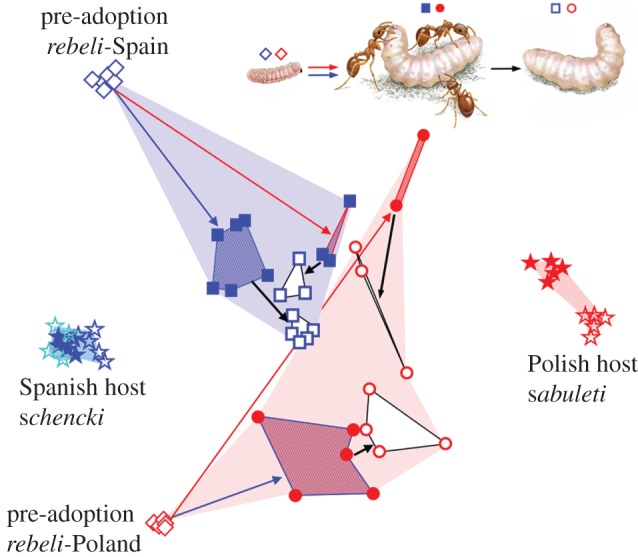
Changes in cuticular hydrocarbon profiles when *Ma. rebeli* larvae are reared with or without different *Myrmica* host species. The non-parametric multi-dimensional scaling plot shows profiles of final-instar butterfly larvae from Spain (blue symbols) and Poland (red) at pre-adoption before encountering ants (diamonds), after 6 weeks (solid squares/circles) with *My. schencki* (blue arrows, boundaries and stippling) and *My. sabuleti* (red arrows, boundaries, stippling), then removal from ants for 5 days (open squares, circles, black arrows and lines), when acquired ant chemicals dissipate and those synthesized by the butterfly accumulate. Stars *My. schencki* (blue), *My. sabuleti* (red); naive test colonies (solid), Spanish (dark), Polish (pale) sites.

Given the multi-functionality of hydrocarbons [39], perfect matches by *Ma. rebeli* secretions to the dissimilar recognition profiles of *My. schencki* or *My. sabuleti* [26] were not expected [27]. Nevertheless, *Ma. rebeli* from each region secreted a distinctive cocktail that mimicked its natural host's signature with increasing likeness (figure 3). After living 6 weeks with laboratory ants, both types of social parasite resembled their natural and artificial hosts significantly more closely than did pre-adoption larvae (*t*_2–3_ =−4.28 to −18.41, *p* = <0.001, see the electronic supplementary material, table S2 for full statistics). However, after 5 days of isolation, the profiles of Spanish *rebeli* reared unnaturally with *My. sabuleti*, and Polish *rebeli* with *My. schencki*, shifted to resemble their natural model more closely (*t*_43_ = 7.38, *p* = < 0.001 and *t*_27_ = 3.26, *p* = 0.003, respectively). In particular, the former lost one compound, tentatively identified as 1-methyl-tricosane (see the electronic supplementary material, table S3), which it had evidently acquired from *My. sabuleti* and which was absent from *My. schencki*, and instead started synthesizing heptacosane and 3-methyl-tricosane, diagnostic hydrocarbons of *My. schencki* which were absent or just detectable on *My. sabuleti*. Similarly, isolated Polish *rebeli* lost dotriacontane and octacosane acquired from *My. schencki* (but undetectable on *My. sabuleti*) and gained docosane, an *n*-alkane characteristic of *My. sabuleti*, but not of *My. schencki.* It is noteworthy that two of these three emerging mimetic hydrocarbons (docosane, 3-methyl-tricosane) synthesized by isolated 7-week-old *Ma. rebeli* larvae were absent from the simpler profiles secreted by pre-adoption larvae. By contrast, individuals reared with their natural host did not change significantly (Spanish *rebeli* with *My. schencki*, *t*_35_ = 0.99, *p* = 0.327) or became less like it (Polish *rebeli* with *My. sabuleti*, *t*_19_ = 5.03, *p* = < 0.001) after isolation.

### (d) Niches of host ants in grassland

We found that *My. schencki* inhabits shorter turf than *My. sabuleti* in the xerotypic grasslands that support *Ma. rebeli* in the Pyrenees (table 1: *My. schencki ≠ My. sabuleti t*_98_ = 3.05, *p* = 0.003; *My. sabuleti ≠ My. scabrinodis t*_29_ = 6.37, *p* = 0.001; *My. scabrinodis ≠ Myrmica rubra* ns). Similarly, as befits the more thermophilous ant, we observed *My. schencki* predominantly in well-grazed swards on skeletal soils in Poland.

**Table 1.** The niches within heterogeneous successional grassland occupied by host and non-host *Myrmica* species that adopted *Maculinea rebeli* larvae in the Pyrenees, Spain, represented by vegetation height (cm, *n* = 314).

	*My. schencki* turf (cm)	*My. sabuleti* turf (cm)	*My. scabrinodis* turf (cm)	*Myrmica rubra* turf (cm)
sward height ± s.e.m.	6.16 ± 0.26	7.60 ± 0.39	17.63 ± 1.50	18.23 ± 0.84

## 4. Discussion

Our results reveal a major difference in the physiology of populations of *Ma. rebeli* in Spain and southeast Poland, enabling each social parasite to infiltrate and exploit a very different *Myrmica* host society—a degree of specialization that makes each incompatible with the other's host species. By contrast, some taxonomists consider *Ma. rebeli* itself to be a mere ecotype of *Ma. alcon* rather than a true species. On current knowledge, the known hosts of these two cuckoo *Maculinea* belong to three distinct groups of *Myrmica* [38]: *rubra* (includes *ruginodis*), *scabrinodis* (includes *sabuleti*) and *lobicornis* (includes *schencki*), of which *Ma. alcon* exploits representatives from the first two groups [9] and *Ma. rebeli* from the second two. Field [40] and pre-adoption chemical [24] evidence suggest that similar exclusive differentiation may have evolved between the main European form of *Ma. alcon* that exploits *My. scabrinodis* and that of Scandinavia and the *Pays-Bas* that is adapted to *My. rubra/ruginodis*. Current molecular techniques compound the confusion, for no wide-scale differentiation was detected between or within *Ma. rebeli* and *Ma. alcon* [20,21], perhaps because current forms of these extreme specialists evolved rapidly in recent millennia [20] and/or very few genes are involved. Unfortunately, lycaenid butterflies in general, and *Maculinea* species in particular, are notoriously difficult to pair in captivity, making large-scale cross-breeding experiments on hybrids exceedingly difficult. Thus, although some morphologists and recent genetic analyses currently recognize one cuckoo species of *Maculinea* (*Ma. alcon*), ecological studies suggest two cryptic species (*Ma. rebeli* and *Ma. alcon*) [9], and our current functional/physiological studies point towards three (possibly four) recent siblings, drawn from the above, exploiting *rubra-*, *scabrinodis*- and *lobicornis-*taxa of *Myrmica*.

Whatever the taxonomic status of each form, all are ill-served by traditional conservation paradigms based on species listing. Like ecospecies [41], each type exploits a resource that occupies a different niche or biotope, and all are threatened by habitat degradation or destruction. In the case of *Ma. rebeli*, *My. schencki* requires more frequently grazed grassland than *My. sabuleti* and considerably more than *My. scabrinodis*. The successful restoration to the UK of *Ma. arion* resulted from creating optimum habitat for its host *My. sabuleti* [16]: similar management would promote *Ma. rebeli* in southeast Poland yet cause population extinctions elsewhere in Poland (figure 1*a*) and in Spain.

Regional host shifts are not unknown in social parasites, especially among cuckoo species [24,40,42]. However, the more different the ecology, physiology, defence and social organization of hosts, the less we consider it probable that the extreme adaptations required to exploit them will be expressed by phenotypes of a single species [43]. Indeed, all six morpho-species of insect social parasite whose ecology, mimicry, host use or genetics have been studied show evidence of cryptic speciation (*Microdon* hoverflies [43], predatory *Maculinea* [15,20,21]) or extreme differentiation (cuckoo *Maculinea*), making it likely that the phenomenon is common among the approximately 10 000 unstudied morpho-species [9] of insect social parasites, many of which are Red Data Book listed [15,25]. Other parasitic systems may be similar, particularly where species' interactions are governed by non-morphological cues such as chemical signalling or resistance [8,10,43]. Thus, while molecular techniques have strengthened the species paradigm by identifying cryptic species among certain types of listed morpho-species [8,10,11], conservationists cannot yet rely on them [12–14] to recognize functionally distinct forms or siblings of extreme specialists which perhaps differ by a single gene or which evolve and disappear over millennia rather than epochs, and yet are among the most interesting and threatened species on the Earth.
